# Carotid artery volumetric measures associate with clinical ten-year cardiovascular (CV) risk scores and individual traditional CV risk factors in rheumatoid arthritis; a carotid-MRI feasibility study

**DOI:** 10.1186/s13075-018-1761-2

**Published:** 2018-12-03

**Authors:** Lesley-Anne Bissell, Bara Erhayiem, Graham Fent, Elizabeth M. A. Hensor, Agata Burska, Helena Donica, Sven Plein, Maya H. Buch, John P. Greenwood, Jacqueline Andrews

**Affiliations:** 1 Leeds Institute of Rheumatic and Musculoskeletal Medicine, University of Leeds, Leeds, UK; 2 NIHR Leeds Biomedical Research Centre, Leeds, UK; 3 Multidisciplinary Cardiovascular Research Centre & The Division of Biomedical Imaging, Leeds Institute of Cardiovascular and Metabolic Medicine, University of Leeds, Leeds, UK; 4 Medical University of Lublin, Lublin, Poland

**Keywords:** Rheumatoid arthritis, Carotid MRI, Atherosclerosis

## Abstract

**Background:**

Common carotid artery intima-media thickness (CIMT), as measured by ultrasound, has utility in stratification of the accelerated cardiovascular risk seen in rheumatoid arthritis (RA); however, the technique has limitations. Carotid magnetic resonance imaging (MRI) is emerging as a useful research tool in the general population, but has yet to be applied in RA populations. Our objectives were to describe the utility of carotid artery MRI (carotid-MRI) in patients with RA in comparison to healthy controls and to describe the association with RA disease phenotype.

**Methods:**

Sixty-four patients with RA and no history of cardiovascular (CV) disease/diabetes mellitus were assessed for RA and CV profile, including homeostasis model assessment-estimated insulin resistance (HOMA-IR) and N-terminal pro-brain natriuretic peptide (NT-proBNP). All underwent carotid-MRI (3 T), and were compared to 24 healthy controls. Univariable analysis (UVA) and multivariable linear regression models (MVA) were used to determine associations between disease phenotype and carotid-MRI measures.

**Results:**

There were no significant differences in carotid arterial wall measurements between patients with RA and controls. Wall and luminal volume correlated with 10-year CV risk scores (adjusted as per 2017 European League Against Rheumatism (EULAR) guidance); rho = 0.33 (*p* = 0.012) and rho = 0.35 (*p* = 0.008), respectively, for Joint British Societies-2 risk score. In UVA, carotid-MRI volumetric measures predominantly were associated with traditional CV risk factors including age, ever-smoking and HOMA-IR (*p* < 0.05). Lower body mass index was associated with wall maximum thickness (*r* = − 0.25 *p* = 0.026). In MVA, age was independently associated with wall volume (B 1.13 (95% CI 0.32, 1.93), *p* = 0.007) and luminal volume (B 3.69 (95% CI 0.55, 6.83, *p* = 0.022), and RA disease duration was associated with luminal volume (B 3.88 (95% CI 0.80, 6.97), *p* = 0.015).

**Conclusions:**

This study demonstrates the utility of carotid-MRI in RA, reporting an association between three-dimensional measures in particular and CV risk scores, individual traditional CV risk factors and RA disease duration. Carotid-MRI in RA is a promising research tool in the investigation of CVD.

**Electronic supplementary material:**

The online version of this article (10.1186/s13075-018-1761-2) contains supplementary material, which is available to authorized users.

## Introduction

Surrogate measures of cardiovascular disease (CVD) in the general population have been applied to patients with rheumatoid arthritis (RA) to investigate their accelerated cardiovascular (CV) risk. Common carotid artery intima-media thickness (CIMT) is one of the best validated surrogate measures of CVD, predicting future CV events in the general population [1]. It has also been shown to be greater in patients with RA [2, 3], being associated with CV events [4], and demonstrating utility in the stratification of CV risk in those with moderate clinical CV risk scores [5].

CIMT is usually measured by ultrasound (US); however, this method is operator dependent, and comparisons between studies are difficult due to varying scanning protocols. Carotid magnetic resonance imaging (carotid-MRI) provides alternative imaging of the carotid artery, with MRI-measured mean wall thickness (MWT), correlating well with US-measured CIMT [6–9]. There is additional interest in MWT given it is the sum of the vessel wall intima, media and also adventitia layer. Evidence suggests the adventitia has an important role in determining CV risk [8]. Specifically, in RA increased expression of inflammatory cytokines in the aortic adventitia of patients with RA undergoing coronary artery bypass grafts has been reported compared to patients without RA [10]. Carotid-MRI measurements are associated with future CV events in the general population [11], but have yet to be measured in RA populations.

This exploratory study aimed to demonstrate the novel use of carotid-MRI in patients with established RA free of known CVD and diabetes mellitus, in comparison to healthy controls, and to describe the association between carotid-MRI measures and RA disease phenotype, to provide insight into the patient phenotype most at risk of CVD.

## Methods

Consecutive patients with RA attending rheumatology clinics between January 2011 and September 2014 at the Leeds Teaching Hospitals National Health Service (NHS) Trust (LTHT) were considered for the IACON (Inflammatory arthritis disease continuum longitudinal) study; REC 09/H1307/98, approved by the Leeds West ethics committee. Patients were eligible if they were between 18 and 80 years old, had had disease for 5 years or more, met the 1987 American College of Rheumatology (ACR) criteria [12] and had no history of CVD (cardiac, peripheral or cerebral) or diabetes mellitus. Healthy controls, with no history of RA or osteoarthritis that affected their mobility, were mainly identified by asking patients with RA to “bring a friend”. Carotid-MRI data from two healthy controls consented into the study “Assessment of myocardial perfusion by magnetic resonance imaging: 3T optimization of acquisition and analysis methods in patients with heart disease” (REC 10/H1307/103, Leeds West ethics committee) were also utilised. Following written informed consent, study participants were invited to undergo a cross-sectional comprehensive CV clinical assessment, fasting blood collection and carotid-MRI.

### Clinical assessment

Demographic data, traditional CV risk factors and for patients with RA, disease phenotype including 3-variable (3v) Disease Activity Score in 28 joints (DAS28) based on C-reactive protein (CRP) protein [13] and Health Assessment Questionnaire Disease Index [14] were recorded on clinical evaluation. Fasting lipid profile and glucose were measured, and rheumatoid factor, anti-cyclic citrullinated peptide antibody, CRP and erythrocyte sedimentation rate were measured in those with RA. Two 10-year cardiovascular risk scores were calculated, given the pilot nature of this work; Framingham (commonly used and therefore understood by a wide audience) and Joint British Societies 2 (in line with our cardiology partnership/network practice), and both were adjusted as per 2010 [15] and 2017 [34] European League Against Rheumatism (EULAR) guidelines. Additional samples were processed and stored for later measurement of NT-proBNP and homeostasis model assessment-estimated insulin resistance (HOMA-IR, fasting insulin (μU/ml) × fasting glucose (mg/dl)/405) [16].

### Carotid-MRI

Non-contrast carotid MRI was performed at 3.0 T (Philips Achieva, Philips, Best, The Netherlands) using a small 10-cm phased-array receiver coil (Philips dStream Flex, Best, The Netherlands). Survey images were used to locate the bifurcation of the common carotid artery into the internal and external carotid artery. These images were used to plan perpendicular, non-breath-held, time-of-flight (ToF) proton density-weighted (PD), T1 and T2 weighted acquisitions of the common carotid artery (10 slices in total, 5 above and 5 below the carotid bifurcation). Voxel sizes for each of these acquisitions were as follows: ToF 0.89 × 0.89 × 3 mm, PD 0.7 × 0.7 × 2 mm, T1 0.7 × 0.7 × 2 mm, T2 0.7 × 0.7 × 2 mm.

Using methodology similar to previously published studies in the general population [17–19] as described subsequently, a cardiology fellow (GF) reviewed and reported the carotid-MR images blinded to all patient details and disease status using post-processing software (QMASS MR 7.5, Medis, Leiden, The Netherlands). An expert cardiovascular MRI-cardiologist (JPG) reviewed a subset of images/contours to ensure all were assessed as expected. A second junior then repeated contouring/measurements for a subset of images to allow for calculation of inter-observer variability.

**Fig. 1:**
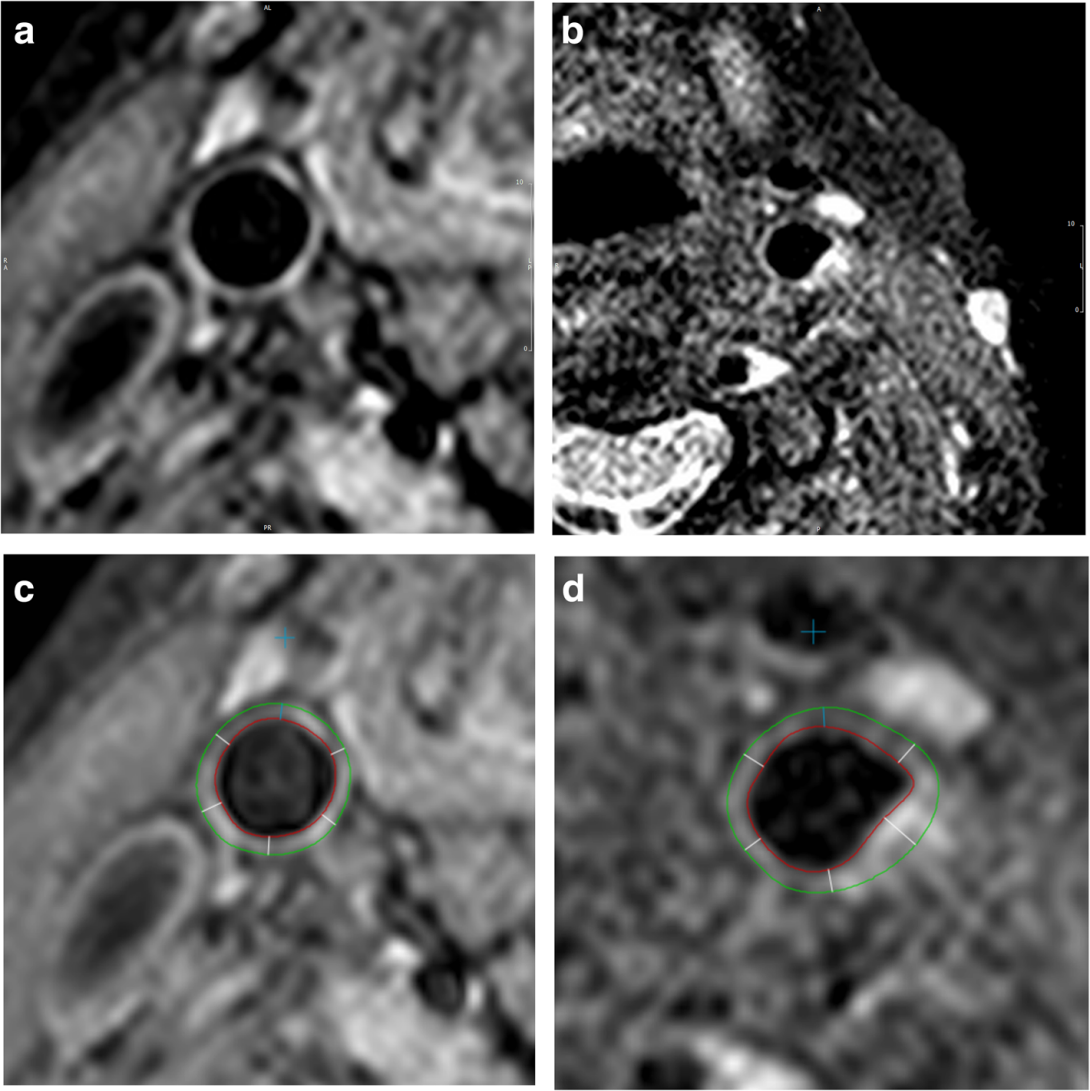
Cross-sectional T1-weighted images of the carotid artery in short-axis views. **a** Normal carotid artery, **b** carotid artery with plaque, **c** normal carotid artery with contours, **d** carotid artery with plaque with contours. Red lines represent the endothelial wall and green lines represent the outer (adventitial) wall of the carotid artery

Cross-sectional T1-weighted images of the right carotid artery were assessed in short-axis views. Slices 2, 4, 6 and 8 mm below the bifurcation of the common carotid artery were identified, and the carotid artery endothelial wall manually contoured on each slice. The outer (adventitial) wall of the artery was also contoured for each slice. Each cross-sectional image was then divided into 6 segments, and the post-processing software derived the minimum and maximum wall thickness, along with MWT of all 24 segments of the carotid wall. Figure 1 illustrates the images acquired and contours drawn. The software then used the contours to calculate the carotid wall mass (wall volume x the density of the tissue (1.05 mg/ul) [20]) for all four slices, allowing calculation of carotid wall volume. Total carotid wall volume was normalised for vessel size (carotid wall volume index) by calculating carotid wall volume/(carotid wall volume + luminal volume).

If the right carotid artery images were of poor quality or did not provide enough slices, the left carotid artery was used for measurement.

### Statistical analysis

The statistical packages SPSS (IBM SPSS Statistics 22) and Stata/IC 13.1 were used (the latter for calculating robust standard errors). Following descriptive analysis, the independent Student *t* test was used to determine differences between patients with RA and controls. Linear regression was used to determine differences when adjusted for age, gender and CV risk factors (defined as hypertension (either history of hypertension or on anti-hypertensive agent), dyslipidaemia (either history of dyslipidaemia, on lipid-lowering medication or total cholesterol/high-density lipoprotein cholesterol (TC/HDL-C) ratio > 6) and ever having smoked). Non-normally distributed variables were log-transformed prior to analysis.

Within the RA group, Spearman’s correlation was used to measure associations between carotid-MRI measures and 10-year CV risk scores, and Pearson’s correlation and univariable linear regression analyses (UVA) were used to measure associations between each of the baseline variables and caroti- MRI measures, using log-transformed values when appropriate. Any variables reported in the literature as associated with carotid-MRI measures or found to be strongly correlated in the preliminary analyses (Pearson’s correlation coefficient > 0.3) were then included in a multivariable linear regression model. When the heteroskedasticity of regression model residuals was not improved by logarithmic transformation of the data, robust standard errors were employed.

Intra and inter-observer variability was tested (between GF and LAB blinded to one another) for assessment of carotid MWT to demonstrate an acceptable coefficient of variance.

In the event of missing serology results the most recent value preceding the visit was carried forward into the data, excluding CRP due to its capacity to vary, or lipid/glucose profile, as a fasting state could not be verified.

## Results

### Study participant characteristics

Of 69 patients with RA and 25 healthy controls that underwent a carotid-MRI scan, 64 with RA and 24 controls had images available to analyse (using left carotid images in 12 and 4 individuals, respectively); 4 data sets were of poor quality and 2 were missing due to image processing errors. Of the patients with RA, 60 patients had data on carotid arterial wall volume measurements; data were missing in 4 patients as the carotid artery bifurcated too low to allow measurement up to 8 mm below the bifurcation.

Table 1 outlines the demographic, CV risk profile and soluble CV biomarkers, and Table 2 describes the RA disease-specific features of those who had carotid artery images available. The mean age (standard deviation) of patients with RA was 59.6 (9.4) years, 70% were female and 94% white. Median (interquartile range) disease duration was 17.3 (10.7, 25.7) years, 84% were seropositive for rheumatoid factor (RF) or anti-citrullinated protein antibodies (ACPA), 82% had erosive disease and 67% were taking biological disease-modifying anti-rheumatic drugs (DMARDs). Patients overall were in remission; median (interquartile range) 3v DAS28 was 2.38 (1.15, 3.25). A significant proportion had CV risk factors, including 33% with known hypertension. The control group were younger (mean (standard deviation) age 51.8 (11.7) years) with fewer (54%) women and fewer CV risk factors. There was little difference in glucose and lipid profile, HOMA-IR or NT-proBNP between patients with RA and controls.

**Table 1:** Study participant characteristics

Variable	Expressed as	Patients with RA	Controls
		*n* = 64	*n* = 24
Demographics			
Age, years	mean, SD	59.6 (9.4) (range 31, 78)	51.8 (11.7) (range 35–80)
Female	*n* (%)	45 (70.3)	13 (54.2)
Ethnicity	*n* (%)	60 (94.0) white	21/22 (95.8) white
CV risk profile			
PMH hypertension	*n* (%)	22 (34.4)	1/21 (4.2)
PMH hypercholesterolaemia	*n* (%)	16 (25.0)	1/21 (4.2)
Smoking status:			
Never	*n* (%)	28 (43.8)	11/21 (52.4)
Ex-smoker		28 (43.8)	8/21 (38.1)
Current		8 (12.5)	2/21 (9.5)
Alcohol intake, units/week	median (IQR)	2 (0, 8) (*n* = 63)	1 (0, 6) (*n* = 21)
FHx premature CVD^a^	*n* (%)	14 (21.9)	4/20 (16.7)
Five or more fruit/vegetables daily intake, days/week	median (IQR)	5 (4, 7) (n = 63)	6 (4, 7) (*n* = 19)
Moderate exercise, mins/week	median (IQR)	40 (0, 156) (*n* = 62)	60 (0, 240) (*n* = 20)
Number of current anti-hypertensives	*n* (%)	8 (12.5) on 1 drug	1/21 (4.8) on 2 drugs
		8 (12.5) on 2 drugs	
		1 (1.6) on 3 drugs	
Current use of statin	*n* (%)	11 (17.2)	1/21 (4.2)
BMI	mean, SD	25.8 (3.3)	25.2 (3.4)
Waist/hip ratio	mean, SD	0.84 (0.08) (n = 62)	0.82 (0.10) (n = 21)
Systolic BP, mmHg	mean, SD	134 (20)	125 (14) (*n* = 23)
Diastolic BP, mmHg	mean, SD	80 (12)	74 (10) (*n* = 23)
Fasting blood tests			
Fasting glucose, mmol/L	mean, SD	4.9 (1.0) (n = 60)	4.7 (0.5) (*n* = 19)
Fasting total cholesterol, mmol/L	mean, SD	5.3 (1.1) (n = 63)	5.1 (1.0) (*n* = 19)
Fasting HDL-C, mmol/L	mean, SD	1.6 (0.4) (*n* = 61)	1.6 (0.4) (*n* = 19)
Fasting LDL-C, mmol/L	mean, SD	3.1 (1.0) (*n* = 61)	3.0 (0.9) (*n* = 19)
Fasting TC/HDL-C ratio	mean, SD	3.4 (1.1) (n = 61)	3.3 (1.0) (*n* = 19)
Fasting triglycerides, mmol/L^b^	geometric mean	1.1 (n = 63)	1.0 (*n* = 19)
HOMA-IR^b^	geometric mean	1.06 (n = 60)^c^	1.13 (*n* = 20)
NT-proBNP, pg/ml^b^	geometric mean	53.3 (*n* = 60)	40.2 (*n* = 20)
Ten-year clinical risk scores			
Framingham:	median (IQR)	14.5 (6.3, 27.9)^d^	4.4 (1.3, 14.4) (*n* = 18)
		14.6 (8.1, 29.1)^e^	
Joint British Societies 2:	median (IQR)	11.9 (4.9, 20.2)^d^	3.9 (0.8, 11.5) (*n* = 18)
		12.0 (6.1, 22.2)^e^	

*BMI* body mass index, *BP* blood pressure, *CVD* cardiovascular disease, *FHx* family history, *HOMA-IR* homeostasis model of assessment of insulin resistance, *LDL-C* low-density lipoprotein cholesterol, *NT-proBNP* N-terminal pro-brain natriuretic peptide, *PMH* past medical history of, *RA* rheumatoid arthritis, *TC/HDL-C* total cholesterol/high-density lipoprotein cholesterol ratio

^a^Defined as first-degree relative with a history of CVD when 60 years old or younger if female, and 55 years old or younger if relative

^b^Variables log transformed prior to analysis

^c^Excluding outlier: 1.01 (*n* = 59)

^d^Adjusted to 2010 European League Against Rheumatism (EULAR) guidelines [15]

^e^Adjusted to 2017 EULAR guidelines [34]

**Table 2:** Disease specific characteristics of patients with Rheumatoid Arthritis

RA phenotype	Data expressed as	Patients with RA, *n* = 68
Disease duration, years	median (IQR)	17.3 (10.7, 25.7) (range 5.4, 43.4)
Early morning stiffness, mins	median (IQR)	13 (0, 45) (*n* = 62)
History of orthopaedic joint surgery	*n* (%)	19 (29.7)
Number of orthopaedic joint surgical episodes	*n* (%)	9 (14.1) - 1 episode
		3 (4.7) - 2 episodes
		5 (7.8) - 3 episodes
		2 (3.1) - 4 episodes
Current use of oral prednisolone	*n* (%)	3 (4.7)
Current use of csDMARD	*n* (%)	52 (81.3)
Number of csDMARDs currently taking	*n* (%)	40 (62.5) taking 1
		8 (12.5) taking 2
		6 (9.4) taking 3
Number of previously tried csDMARDs	median (IQR)	2 (1, 3) (range 0, 7)
Current use of biological DMARD	*n* (%)	43 (67.2)
Current TNFI users		19 (29.7)
Current rituximab users		21 (32.8)
Current tocilizumab users		2 (3.1)
Current abatacept user		1 (1.6)
Number of treatment cycles in current RTX users	median (IQR)	4 (3, 5.5) (range 2, 9)
Number of previously tried biological DMARDs	median (IQR)	0 (0, 1)
Patient general health VAS	median (IQR)	31 (16, 53) (*n* = 59)
TJC28	median (IQR)	2 (0, 6)
SJC28	median (IQR)	0 (0, 1)
HAQ-DI	median (IQR)	1.50 (0.53, 2.00) (*n* = 60)
3-variable DAS28-CRP	median (IQR)	2.38 (1.15, 3.25)
Erosions on hands/feet x-ray	*n* (%)	50/61 (82)
CRP (mg/L)	median (IQR)	0 (0, 8.3)
ESR (mm/h)	median (IQR)	14 (6, 28) (*n* = 59)
RF positive (≥ 40 iu/ml)	*n* (%)	44 (68.8)
ACPA positive (≥ 10 U/ml)	*n* (%)	50/63 (79.4)

*ACPA* anti-citrullinated peptide antibody, *CRP* C-reactive protein, *csDMARDs* conventional synthetic DMARDs, *DAS28-CRP* 28-joint disease activity score based on CRP, *DMARDs* disease-modifying anti-rheumatic drugs, *ESR* erythrocyte sedimentation rate, *HAQ-DI* Health Assessment Questionnaire-Disability Index, *RF* rheumatoid factor, *RTX* rituximab, *SJC* swollen joint count, *TJC* tender joint count, *VAS* visual assessment score

### Carotid-MRI results

There were no significant differences between patients with RA and controls in carotid arterial wall measurements, including MWT, maximum wall thickness, wall volume, luminal volume, wall volume index and wall volume indexed/body surface area (BSA) (see Table 3).

**Table 3:** Carotid artery magnetic resonance imaging measures in study participants

Carotid artery variable	Patients with RA, *n* = 64	Controls, *n* = 24	Unadjusted differences in RA group from controls		Mean difference adjusted for age and sex		Mean difference adjusted for age, sex and CV risk factors^b^	
			Mean (95% CI)	*P* value	B (95% CI)	*P* value	B (95%)	*P* value
Mean wall thickness, mm	1.051 (0.125)	1.029 (0.129)	0.022 (− 0.038, 0.082)	0.460	0.002 (− 0.060, 0.064)	0.940	−0.009 (− 0.079, 0.060)	0.790
Minimum wall thickness, mm	0.747 (0.096)	0.718 (0.107)	0.029 (− 0.019, 0.076)	0.230	0.013 (− 0.036, 0.063)	0.591	0.000 (− 0.056, 0.056)	0.990
Maximum wall thickness, mm	1.498 (0.330)	1.530 (0.311)	− 0.031 (− 0.186, 0.123)	0.688	− 0.050 (− 0.217, 0.118)	0.556	− 0.045 (− 0.237, 0.148)^c^	0.646
Wall volume^a^, ul	234.528 (45.187) (*n* = 60)	225.613 (46.751)	8.914 (−13.010, 30.839)	0.421	−5.053 (−26.236, 16.131)	0.636	−5.479 (−28.979, 18.021)	0.644
Luminal volume^a^, ul	383.408 (116.277) (n = 60)	368.753 (105.625)	14.655 (−39.82, 69.135)	0.594	−0.430 (−55.585, 54.726)	0.988	−8.792 (−71.799, 54.215)	0.782
Wall volume indexed^a^	0.385(0.046) (n = 60)	0.385 (0.040)	0.001 (− 0.020, 0.022)	0.930	0.001 (−0.023, 0.024)	0.963	0.001 (−0.026, 0.027)	0.952
Wall volume indexed/BSA	0.216 (0.036) (n = 60)	0.221 (0.032)	−0.005 (− 0.021, 0.012)	0.567	− 0.11 (− 0.029, 0.006)	0.201	−0.008 (− 0.028, 0.012)	0.430

*CV* cardiovascular, *BSA* body surface area, *RA* rheumatoid arthritis

^a^Of 6 mm length of carotid artery. Carotid wall volume indexed calculated by carotid wall volume/(carotid wall volume + luminal volume)

^b^CV risk factors defined as: hypertension (history of hypertension or anti-hypertensive agent), dyslipidaemia (history of dyslipidaemia, on lipid-lowering medication or total cholesterol/high-density lipoprotein cholesterol ratio > 6), ever smoked and family history of premature cardiovascular disease

^c^Heteroskedasticity of residuals therefore robust standard errors employed to compensate

### Association with disease phenotype

In patients with RA, carotid wall volume and luminal volume correlated well with 10-year CV risk scores (adjusted as per 2010 [15] and 2017 [34] EULAR guidelines). The 2010 adjustments appeared to perform better; the carotid wall and luminal volume Spearman correlation coefficients for the 2010 adjusted Joint British Societies 2 risk scores were 0.37 (*p* = 0.005) and 0.43 (*p* = 0.001), respectively, compared to the 2017 adjusted Joint British Societies 2 risk scores (rho = 0.33 (*p* = 0.012) and rho = 0.35 (*p* = 0.008), respectively) (see Table 4).

**Table 4:** The association between carotid wall variables in patients with RA and 10-year cardiovascular risk scores (adjusted as per 2010 and 2017 EULAR guidelines)

Variable	Adjusted 10-year Framingham cardiovascular risk score						Adjusted 10-year Joint British Societies 2 cardiovascular risk score					
	2010 EULAR guidelines			2017 EULAR guidelines			2010 EULAR guidelines			2017 EULAR guidelines		
	Rho	95% CI	*P* value	Rho	95% CI	*P* value	Rho	95% CI	*P* value	Rho	95% CI	*P* value
Mean wall thickness	0.10	−0.16, 0.34	0.451	0.10	− 0.15, 0.35	0.426	0.09	−0.17, 0.33	0.514	0.09	−0.07, 0.33	0.517
Minimum wall thickness	0.12	−0.14, 0.36	0.368	0.13	−0.13, 0.37	0.335	0.13	−0.13, 0.37	0.322	0.13	−0.12, 0.37	0.309
Maximum wall thickness	0.06	−0.19, 0.31	0.622	0.05	−0.20, 0.30	0.684	0.04	−0.21, 0.29	0.734	0.03	−0.23, 0.28	0.848
Wall volume^a^	0.38	0.13, 0.58	0.004	0.34	0.09, 0.53	0.009	0.37	0.12, 0.58	0.005	0.33	0.08, 0.54	0.012
Luminal volume^a^	0.43	0.19, 0.62	0.001	0.35	0.10, 0.56	0.007	0.43	0.19, 0.62	0.001	0.35	0.10, 0.56	0.008
Wall volume indexed^a^	− 0.22	−0.46, 0.04	0.094	−0.17	− 0.42, 0.09	0.198	− 0.23	−0.46, 0.04	0.088	−0.19	− 0.43, 0.08	0.167
Wall volume indexed/BSA	−0.24	−0.47, 0.02	0.068	−0.21	− 0.45, 0.05	0.117	− 0.26	−0.48, 0.01	0.055	−0.23	− 0.46, 0.04	0.093

Number of observations = 61 unless otherwise stated. CI (confidence intervals) are of the correlation coefficient

*RA* rheumatoid arthritis, *Rho* Spearman correlation coefficient, *EULAR* European League Against Rheumatism, *BSA* body surface area

^a^Number of observations = 57

No variables (traditional CV risk factors or RA disease-specific factors) were associated with carotid MWT (see Additional file 1: Table S1). A history of smoking and lower BMI correlated with higher maximum wall thickness (Pearson’s correlation coefficient *r* = 0.25 *p* = 0.049, *r* = − 0.25 *p* = 0.026) (see Additional file 1: Table S2); given *r* < 0.3, only age and gender were added to the multivariable analysis model (MVA) and no independent association was revealed.

In unadjusted analyses, increasing age (*r* = 0.35, *p* = 0.007) and a history of smoking (*r* = 0.33, *p* = 0.010) were associated with carotid wall volume, although only age remained independently associated in the MVA (B 1.13 (95% CI 0.32, 1.93), *p* = 0.007, *R*^2^ = 0.245 (see Table 5). Increasing age (*r* = 0.37, *p* = 0.004), HOMA-IR (*r* = 0.30, *p* = 0.024) and RA disease duration (*r* = 0.34, *p* = 0.007) were associated with carotid luminal volume, although the association with HOMA-IR was lost with the exclusion of the high outlier. Age and RA disease duration remained independently associated in the MVA (age, B 3.69 (95% CI 0.55, 6.83) *p* = 0.022, RA disease duration; B 3.88 (95% CI 0.80, 6.97) *p* = 0.015, R^2^ = 0.257 (see Additional file 1: Table S3).

**Table 5:** Regression analysis of variables associated with carotid wall volume in patients with RA

Variable	Carotid wall volume (ul)				
	Univariable analysis (*n* = 60, unless otherwise stated)			Multivariable analysis^b^	
				*R*^2^ = 0.250, *n* = 60	
	Correlation coefficient	B (95% CI)	*P* value	B (95% CI)	*P* value
Age^a^	0.35	1.63 (0.47, 2.79)	0.007	1.13 (0.32, 1.93)	0.007
Male gender^a^	0.25	24.49 (0.01, 48.98)	0.050	15.99 (−10.62, 42.60)	0.234
Systolic blood pressure	0.13	0.28 (−0.29, 0.85)	0.329	–	–
Ever smoked	0.33	29.71 (7.26, 52.16)	0.010	19.43 (−1.48, 40.34)	0.068
Body mass index	−0.09	−1.16 (−4.66, 2.34)	0.508	–	–
Waist/hip circumference	0.18	100.40 (−43.20, 244.001) (*n* = 58)	0.167	–	–
TC/HDL-C	−0.07	−2.92 (−14.74, 8.90) (*n* = 57)	0.622	–	–
HOMA-IR	0.09	1.66 (−0.61, 3.93)^b^ (*n* = 56)^c^	0.523	–	–
NT-proBNP	−0.09	−0.08 (− 0.33, 0.16) (n = 56)	0.499	–	–
RA disease duration	0.31	1.36 (0.25, 2.46)	0.017	0.78 (−0.24, 1.79)	0.131
3-variable DAS28	−0.021	−0.75 (− 10.07, 8.57)	0.872	–	–
ACPA	0077	8.58 (−21.004, 38.18) (n = 59)	0.564	–	–
HAQ-DI	0.07	3.78 (− 11.98, 19.54) (*n* = 57)	0.633	–	–
History of joint surgery	−0.02	−1.76 (−27.45, 23.94)	0.892	–	–
Current use of biological DMARD	−0.02	−1.582 (−25.84, 23.79)	0.905	–	–

*ACPA* anti-citrullinated peptide antibody, *CI* confidence intervals, *CRP* C-reactive protein, *DAS28* 28-joint disease activity score, *DMARD* disease-modifying anti-rheumatic drug, *HAQ-DI* Health Assessment Questionnaire-Disability Index, *HOMA-IR* homeostasis model of assessment of insulin resistance, *MWT* mean wall thickness, *NT-proBNP* N-terminal pro-brain natriuretic peptide, *TC/HDL-C* total cholesterol/high-density lipoprotein cholesterol ratio

^a^Variable entered into linear regression model as associated with carotid wall volume in the literature

^b^Heteroskedasticity of residuals therefore robust standard errors employed to compensate

^c^Excluding high outlier: correlation coefficient − 0.07 B (95% CI) − 3.82 (− 15.45, 7.82) *p* = 0.513

A history of previous joint surgery and increasing HOMA-IR was associated with lower carotid wall volume index (*r* = − 0.26 *p* = 0.043 and *r* = − 0.28 *p* = 0.035, respectively), again the association with HOMA-IR was lost with the exclusion of the outlier. Only age and gender were included in the MVA and no independent association was determined (see Additional file 1: Table S4).

### Intra-observer and inter-observer variability

Cardiology fellow GF re-analysed 10 carotid images. The mean difference for intra-observer variability was 0.021 mm (95% CI − 0.012, 0.054) and the limits of agreement were − 0.072 to 0.113 mm. The coefficient of variance was 3.66%. The mean difference for inter-observer assessments (between fellow GF and LAB, using 10 images) was 0.032 mm (95% CI − 0.075, 0.010) and the limits of agreement were − 0.088 to 0.152 mm. The coefficient of variance was 4.93%.

## Discussion

This is the largest study to date using carotid-MRI to describe carotid artery morphology in detail in comparison to healthy controls and the association with RA disease phenotype. No significant differences were seen in carotid wall measurements between those with RA and healthy controls. However, carotid vessel wall and luminal volumes correlated well with 10-year CV risk scores, and were associated with traditional CV risk factors, along with RA disease duration.

Carotid-MRI in RA has been reported in one recent study. Skeoch et al. described late gadolinium-enhanced carotid-MRI in 15 patients with RA and more than 2 mm of plaque on carotid ultrasound. They reported more calcification in those with RA compared to five controls despite similar plaque volume, remodelling index and lipid-rich necrotic core measures. Additionally, 12 of 13 of the patients with RA had evidence of inflammation on (18)F-fluorodeoxyglucose positron emission tomography scanning, correlating with highly sensitive (hs)CRP, but not IL-6 levels or the DAS-28 [21]. Our study reported an association with traditional CV risk factors and RA disease duration, but not CRP or disease activity. However, overall our patients were in remission (median 3-variable DAS28 = 2.38) and those in the study by Skeoch et al. overall had moderately active disease (mean DAS28 = 4.62), which may explain this difference. Additionally, there are recent ultrasound data demonstrating no progression of CIMT over time in those in remission or with low disease activity [22].

Carotid-MRI studies have previously demonstrated an association with traditional CV risk factors in the general population. The largest report from the “Atherosclerosis risk in communities” study (*n* = 1670), revealed carotid wall volume, thickness, and normalised wall index were positively associated with lipids, including total cholesterol, low-density lipoprotein cholesterol (LDL-C), and apo-lipoprotein B [23]. Li et al. reported that being male and being older were associated with MWT and maximum wall thickness in 196 study participants without CVD [24]. A smaller study assessing those with obstructive sleep apnoea (*n* = 42) found an association between maximum wall thickness and waist/hip circumference, mean arterial blood pressure, Framingham risk scores, HDL-C, HOMA-IR, insulin and CRP [25].

The strongest associations with CV risk in our study were observed in the three-dimensional (3-D) volume measurements, which is an advantage of using carotid-MRI over ultrasound. The 3-D outcome measures could provide a more accurate representation of the carotid wall, given that they utilise more data in their calculation. Carotid volumes are of particular interest, as they could reflect carotid arterial wall remodelling; in particular, positive remodelling (or “compensatory enlargement”, seen in the initial stages of atherosclerosis) where a compensatory increase in lumen diameter occurs, together with an increase in wall thickness, in an effort to reduce the development of luminal stenosis. Positive remodelling has been associated with hypertension [26], and also “softer plaque”, i.e. less calcification, and greater plaque instability [26, 27]. Interestingly, Van Sijl et al., using ultrasound, determined that patients with RA (*n* = 96) compared to controls (*n* = 274), despite having similar CIMT values, had a larger vessel lumen diameter, increased adventitia and greater wall stress and tension, after adjustment for CV risk factors [28], suggestive of a positive remodelling process.

### Limitations

There are many limitations associated with this study as outlined below. No widely recognised protocol for carotid-MRI reporting is currently available, and although our methodology mirrored previously published methods, this is an area in need of further study, including concordance between right and left carotid artery measurements. In addition, given the small differences in carotid wall thickness, our inter-observer and intra-observer variability limits of agreement are relatively wide and any future larger study would need to be appropriately powered to be meaningful. The variability in measurement may ultimately prove too wide for carotid-MRI to be a valid outcome measure. This cross-sectional study was also unable to quantify the burden of inflammation to which patients with RA were exposed over their disease duration. The cohort had established severe disease (lengthy disease duration, high proportion of seropositivity/erosions); therefore, although they were in remission, we feel the total burden of inflammation was likely significant as many had required multiple (including biological) disease-modifying anti-rheumatic drugs and orthopaedic surgery. However, the lack of association between carotid imaging measurements and RA disease activity/inflammation may be due to the smaller representation of those with high disease activity in our study population. The effect of acute active RA disease compared to chronic active RA, and those with previous high disease activity but now in remission needs evaluating further with larger studies to make any firm conclusions. We acknowledge that any further study would also require matching for age/sex/CV risk factors to have the greatest chance of detecting any real difference, if present at all, between those with RA and those without. Comparing carotid-MRI measures to US-measured CIMT would also help determine the role/applicability of carotid-MRI in this field both as a research tool and for clinical use in CV screening. Its role in screening is especially important given the relative lack of access to, and greater cost/expertise required, for carotid-MRI and the conflicting evidence supporting inclusion of carotid artery structural measures in CV risk scores in the general population [29, 30].

### Future research agenda

In addition to the needs already discussed, larger cross-sectional studies and prospective longitudinal studies are required to validate the utility of carotid-MRI in RA. The use of contrast to characterise carotid plaque further, differentiating unstable from stable plaques, lipid-rich necrotic cores from intra-plaque haemorrhage [31], combined with positron emission tomography imaging [32] could potentially help provide information on the pathophysiology of CVD in RA and on identifying those at risk of CVD. Our study also provides supportive evidence for strategic management of modifiable traditional CV risk factors in reducing CV morbidity and mortality in RA; an important message given that the management of such patients remains suboptimal [33].

## Conclusions

This study has demonstrated the utility of carotid-MRI in patients with RA, with initial results suggesting an association with traditional CV risk factors, particularly with 3-D carotid-MRI measures. In addition to reinforcing the need to manage traditional CV risk factors effectively in RA, this study suggests a possible alternative surrogate outcome measure for evaluating CV risk in RA. Given the inclusion of the adventitia in carotid-MRI measures, future studies should look to quantify any advantage over US-measured CIMT in the prediction of future CVD, and investigate the utility of additional imaging techniques, such as positron emission tomography, for clinical risk stratification and elucidating the underlying pathophysiology.

## Additional file

Additional file 1: **Table S1** Regression analysis of variables associated with mean carotid wall thickness in patients with RA. **Table S2** Regression analysis of variables associated with maximum carotid wall thickness in patients with RA. **Table S3** Regression analysis of variables associated with carotid luminal volume in patients with RA. **Table S4** Regression analysis of variables associated with carotid wall volume index in patients with RA. (DOCX 28 kb)

## Abbreviations

ACPA: Anti-citrullinated protein antibodies
BMI: Body mass index
BSA: Body surface area
CI: Confidence interval
CIMT: Common carotid artery intima-media thickness
CRP: C-reactive protein
CV: Cardiovascular
CVD: Cardiovascular disease
DAS: Disease activity score
DMARD: Disease-modifying anti-rheumatic drug
EULAR: European League Against Rheumatism
HOMA-IR: Homeostasis model assessment-estimated insulin resistance
IL: Interleukin
LDL-C: Low-density lipoprotein cholesterol
LV: Left ventricular
MRI: Magnetic resonance imaging
MWT: Mean wall thickness
NHS: National Health Service
NT-proBNP: N-terminal pro-brain natriuretic
RA: Rheumatoid arthritis
RF: Rheumatoid factor
TC/HDL-C: Total cholesterol/high-density lipoprotein cholesterol ratio
