# Supplementary material for: Carotid artery volumetric measures associate with clinical ten-year cardiovascular (CV) risk scores and individual traditional CV risk factors in rheumatoid arthritis; a carotid-MRI feasibility study
Source: Arthritis Res Ther. 2018 Dec 3;20:266. doi: 10.1186/s13075-018-1761-2 (PMC6278168; doi:10.1186/s13075-018-1761-2)
Supplement: Supplementary file 1 — Table S1 Regression analysis of variables associated with mean carotid wall thickness in patients with RA. Table S2 Regression analysis of variables associated with maximum carotid wall thickness in patients with RA. Table S3 Regression analysis of variables associated with carotid luminal volume in patients with RA. Table S4 Regression analysis of variables associated with carotid wall volume index in patients with RA. (DOCX 28 kb) [file 13075_2018_1761_MOESM1_ESM.docx]

## Additional file 1

Table S1 to S4 describe the regression analysis of disease phenotype with carotid-MRI measures in Rheumatoid Arthritis (RA).

Table S1: Regression analysis of variables associated with mean carotid wall thickness in patients with RA.

| **Variable** | **Mean wall thickness (mm)** | | | | |
| --- | --- | --- | --- | --- | --- |
|  | **Univariable analysis**  **(n =64, unless otherwise stated)** | | | **Multivariable analysis**  **R^2^=0.031, n=64** | |
|  | **Correlation coefficient** | **B (95% CI)** | ***P* value** | **B (95% CI)** | ***P* value** |
| Age* | 0.16 | 0.00 (-0.001, 0.01) | 0.209 | 0.002 (-0.001, 0.005) | 0.235 |
| Male gender* | 0.09 | 0.02 (-0.05, 0.09) | 0.491 | 0.02 (-0.05, 0.09) | 0.568 |
| Systolic blood pressure | -0.03 | 0.00 (-0.002, 0.001) | 0.795 | - |  |
| Ever smoked | 0.21 | 0.05 (-0.01, 0.11) | 0.099 | - |  |
| Body mass index | -0.12 | -0.01 (-0.01, 0.01) | 0.328 | - |  |
| Waist/hip circumference | 0.11 | 0.17 (-0.22, 0.56) (n=62) | 0.393 | - |  |
| TC/HDL-C | -0.11 | -0.01 (-0.04, 0.02) (n=61) | 0.422 | - |  |
| HOMA-IR | -0.14 | -0.01 (-0.02, 0.01) (n=60)^ | 0.303 | - |  |
| NT-proBNP | 0.01 | 0.00 (-0.001, 0.001) (n=60) | 0.961 | - |  |
| RA disease duration | 0.08 | 0.001 (-0.002, 0.004) | 0.528 | - |  |
| 3 variable DAS28 | -0.09 | -0.008 (-0.03, 0.02) | 0.488 | - |  |
| ACPA | 0.02 | 0.01 (-0.07, 0.09) (n=63) | 0.868 | - |  |
| HAQ-DI | -0.02 | -0.004 (-0.05, 0.04) (n=60) | 0.859 | - |  |
| History of joint surgery | -0.18 | -0.05 (-0.12, 0.02) | 0.150 | - |  |
| Current use of biological DMARD | 0.03 | 0.01 (-0.06, 0.08) | 0.798 | - |  |

ACPA, anti-citrullinated peptide antibody; CI, confidence intervals; CRP, C-reactive protein; DAS28CRP, 28 joint disease activity score; DMARD, disease modifying anti-rheumatic drug; HAQ-DI, health assessment questionnaire-disability index; HOMA-IR, homeostasis model of assessment of insulin resistance; MWT, mean wall thickness; NT-proBNP, N-terminal pro-brain natriuretic peptide; TC/HDL-C, total cholesterol/high-density lipoprotein cholesterol ratio

*variable entered into linear regression model as associated with MWT in the literature

^Excluding high outlier: Correlation coefficient -0.169 B (95% CI) -0.024 (-0.062, 0.013) p=0.200

Table S2: Regression analysis of variables associated with maximum carotid wall thickness in patients with RA.

| **Variable** | **Maximum wall thickness (mm)** | | | | |
| --- | --- | --- | --- | --- | --- |
|  | **Univariable analysis**  **(n =64, unless otherwise stated)** | | | **Multivariable analysis****  **R^2^=0.001, n=64** | |
|  | **Correlation coefficient** | **B (95% CI)** | ***P* value** | **B (95% CI)** | ***P* value** |
| Age* | 0.04 | 0.001 | 0.783 | 0.001 (-0.009, 0.012) | 0.820 |
| Male gender* | 0.01 | 0.01 (-0.18, 0.19) | 0.947 | 0.004 (-0.193, 0.200) | 0.971 |
| Systolic blood pressure | -0.12 | -0.002 (-0.006, 0.002)** | 0.346 | - | - |
| Ever smoked | 0.25 | 0.16 (0.001, 0.33) | **0.049** | - | - |
| Body mass index | -0.25 | -0.02 (-0.05, -0.003)** | **0.026** | - | - |
| Waist/hip circumference | 0.03 | 0.12 (-0.92, 1.16) (n=62) | 0.815 | - | - |
| TC/HDL-C | -0.13 | -0.04 (-0.02, 0.04)** (n=61) | 0.289 | - | - |
| HOMA-IR | -0.13 | -0.02 (-0.03, -0.004)** (n=60)^ | 0.316 | - | - |
| NT-proBNP | -0.07 | -0.0005 (-0.002, 0.001) (n=60) | 0.574 | - | - |
| RA disease duration | 0.06 | 0.002 (-0.006, 0.010) | 0.616 | - | - |
| 3 variable DAS28 | 0.007 | 0.002 (-0.061, 0.065) | 0.959 | - | - |
| ACPA | 0.01 | 0.01 (-0.20, 0.21) (n=63) | 0.951 | - | - |
| HAQ-DI | 0.02 | 0.01 (-0.10, 0.12) (n=60) | 0.893 | - | - |
| History of joint surgery | -0.19 | -0.13 (-0.31, 0.05) | 0.141 | - | - |
| Current use of biological DMARD | -0.08 | -0.05 (-0.23, 0.12) | 0.547 | - | - |

ACPA, anti-citrullinated peptide antibody; CI, confidence intervals; CRP, C-reactive protein; DAS28CRP, 28 joint disease activity score; DMARD, disease modifying anti-rheumatic drug; HAQ-DI, health assessment questionnaire-disability index; HOMA-IR, homeostasis model of assessment of insulin resistance; MWT, mean wall thickness; NT-proBNP, N-terminal pro-brain natriuretic peptide; TC/HDL-C, total cholesterol/high-density lipoprotein cholesterol ratio

*variable entered into linear regression model as associated with maximum wall thickness in the literature

** heteroskedasticity of residuals therefore robust standard errors employed to compensate

^Excluding high outlier: Correlation coefficient -0.12 B (95% CI) -0.05 (-0.12, 0.03) p=0.217

**Table S3: Regression analysis of variables associated with carotid luminal volume in patients with RA.**

| **Variable** | **Carotid luminal volume (ul)** | | | | |
| --- | --- | --- | --- | --- | --- |
|  | **Univariable analysis**  **(n =60, unless otherwise stated)** | | | **Multivariable analysis****  **R^2^=0.257, n=55** | |
|  | **Correlation coefficient** | **B (95% CI)** | ***P* value** | **B (95% CI)** | ***P* value** |
| Age* | **0.37** | **4.43 (1.47, 7.39)** | **0.004** | **3.69 (0.55, 6.83)** | **0.022** |
| Male gender* | 0.25 | 62.98 (-0.04, 125.99) | 0.050 | 28.19 (-33.97, 90.20) | 0.367 |
| Systolic blood pressure | 0.23 | 1.29 (-1.15, 2.78) | 0.078 | - | - |
| Ever smoked | 0.21 | 48.65 (-11.16, 108.46) | 0.109 | - | - |
| Body mass index | -0.001 | -0.04 (-9.08, 9.00) | 0.993 | - | - |
| Waist/hip circumference | 0.13 | 181.91 (-193.44, 557.25 (n=58) | 0.336 | - | - |
| TC/HDL-C | -0.01 | -1.21 (-31.37, 28.96) (n=57) | 0.936 | - | - |
| HOMA-IR | **0.30** | **14.92 (2.08, 27.77) (n=56)^** | **0.024** | - | - |
| NT-proBNP | -0.11 | -0.27 (-0.90, 0.37) (n=56) | 0.407 | - | - |
| RA disease duration | **0.34** | **3.91 (1.10, 6.72)** | **0.007** | **3.88 (0.80, 6.97)** | **0.015** |
| 3 variable DAS28 | 0.092 | 8.42 (-15.46, 21.30) | 0.483 | - | - |
| ACPA | 0.06 | 15.93 (-60.57, 92.43) (n=59) | 0.678 | - | - |
| HAQ-DI | 0.14 | 21.34 (-18.59, 60.96) (n=57) | 0.285 | - | - |
| History of joint surgery | 0.16 | 41.001 (-24.25, 106.25) | 0.213 | - | - |
| Current use of biological DMARD | -0.08 | -20.88 (-85.80, 44.04) | 0.522 | - | - |

ACPA, anti-citrullinated peptide antibody; CI, confidence intervals; CRP, C-reactive protein; DAS28CRP, 28 joint disease activity score; DMARD, disease modifying anti-rheumatic drug; HAQ-DI, health assessment questionnaire-disability index; HOMA-IR, homeostasis model of assessment of insulin resistance; MWT, mean wall thickness; NT-proBNP, N-terminal pro-brain natriuretic peptide; TC/HDL-C, total cholesterol/high-density lipoprotein cholesterol ratio

*variable entered into linear regression model as associated with carotid wall volume in the literature

^Excluding high outlier: Correlation coefficient 0.08 B 11.11 (95% CI -25.03, 47.24) p=0.540

**Table S4: Regression analysis of variables associated with carotid wall volume index in patients with RA.**

| **Variable** | **Carotid wall volume index** | | | | |
| --- | --- | --- | --- | --- | --- |
|  | **Univariable analysis**  **(n=60, unless otherwise stated)** | | | **Multivariable analysis**  **R^2^=0.026, n=60** | |
|  | **Correlation coefficient** | **B (95% CI)** | ***P* value** | **B (95% CI)** | ***P* value** |
| Age* | -0.14 | -0.001 (-0.002, 0.001) | 0.278 | -0.001 (-0.002, 0.001) | 0.316 |
| Male gender* | -0.09 | -0.01 (-0.0, 0.02) | 0.490 | -0.01 (-0.03, 0.02) | 0.572 |
| Systolic blood pressure | -0.17 | 0.0004 (-0.001, 0.0002) | 0.199 | - |  |
| Ever smoked | 0.01 | 0.001 (-0.023, 0.025) | 0.957 | - |  |
| Body mass index | -0.08 | -0.001 (-0.005, 0.002) | 0.530 | - |  |
| Waist/hip circumference | 0.02 | 0.01 (-0.14, 0.16) (n=58) | 0.860 | - |  |
| TC/HDL-C | -0.09 | -0.004 (-0.015, 0.008) | 0.520 | - |  |
| HOMA-IR | -0.28 | -0.01 (-0.01, -0.0004) (n=56)^ | **0.035** | - |  |
| NT-proBNP | 0.08 | 0.00007 (-0.0002, 0.0003) (n=56) | 0.559 | - |  |
| RA disease duration | -0.25 | -0.001 (-0.002, 0.000004) | 0.051 | **-** |  |
| 3 variable DAS28 | -0.158 | -0.006 (-0.015, 0.004) | 0.226 | - |  |
| ACPA | -0.06 | -0.01 (-0.04, 0.02) (n=59) | 0.651 | - |  |
| HAQ-DI | -0.15 | -0.01 (-0.02, 0.01) (n=57) | 0.252 | - |  |
| History of joint surgery | -0.26 | -0.03 (-0.05, -0.001) | **0.043** | - |  |
| Current use of biological DMARD | 0.06 | 0.01 (-0.02, 0.03) | 0.679 | - |  |

ACPA, anti-citrullinated peptide antibody; CRP, C-reactive protein; DAS28CRP, 28 joint disease activity score; DMARD, disease modifying anti-rheumatic drug; HAQ-DI, health assessment questionnaire-disability index; HOMA-IR, homeostasis model of assessment of insulin resistance; MWT, mean wall thickness; NT-proBNP, N-terminal pro-brain natriuretic peptide; S'=Peak systolic strain rate; TC/HDL-C, total cholesterol/high-density lipoprotein cholesterol ratio

*variable entered into linear regression model as associated with carotid wall volume index in the literature

^Excluding high outlier: Correlation coefficient -0.15 B (95% CI) -0.01 (-0.02, 0.01) p=0.262
